# Endoscopic Resection of Skull Base Teratoma in Klippel-Feil Syndrome through Use of Combined Ultrasonic and Bipolar Diathermy Platforms

**DOI:** 10.1155/2017/6384586

**Published:** 2017-01-04

**Authors:** Justin A. Edward, Alkis J. Psaltis, Ryan A. Williams, Gregory W. Charville, Robert L. Dodd, Jayakar V. Nayak

**Affiliations:** ^1^Division of Rhinology, Department of Otolaryngology-Head and Neck Surgery, Stanford University School of Medicine, Stanford, CA 94305, USA; ^2^Department of Surgery-Otorhinolaryngology, Head and Neck Surgery, University of Adelaide, Adelaide, SA, Australia; ^3^Department of Pathology, Stanford University School of Medicine, Stanford, CA 94305, USA; ^4^Department of Neurosurgery, Stanford University School of Medicine, Stanford, CA 94305, USA

## Abstract

Klippel-Feil syndrome (KFS) is associated with numerous craniofacial abnormalities but rarely with skull base tumor formation. We report an unusual and dramatic case of a symptomatic, mature skull base teratoma in an adult patient with KFS, with extension through the basisphenoid to obstruct the nasopharynx. This benign lesion was associated with midline palatal and cerebral defects, most notably pituitary and vertebrobasilar arteriolar duplications. A multidisciplinary workup and a complete endoscopic, transnasal surgical approach between otolaryngology and neurosurgery were undertaken. Out of concern for vascular control of the fibrofatty dense tumor stalk at the skull base and need for complete teratoma resection, we successfully employed a tissue resection tool with combined ultrasonic and bipolar diathermy to the tumor pedicle at the sphenoid/clivus junction. No CSF leak or major hemorrhage was noted using this endonasal approach, and no concerning postoperative sequelae were encountered. The patient continues to do well now 3 years after tumor extirpation, with resolution of all preoperative symptoms and absence of teratoma recurrence. KFS, teratoma biology, endocrine gland duplication, and the complex considerations required for successfully addressing this type of advanced skull base pathology are all reviewed herein.

## 1. Introduction

Klippel-Feil syndrome (KFS) is a rare, skeletal bone disorder primarily associated with any form of congenital fusion anomaly of the cervical vertebrate. The classic triad in KFS consists of brevicollis, low posterior hairline, and severe restriction of neck motion due to congenital cervical vertebral fusion, recently linked to mutations in the GDF3 and GDF6 genes [1]. Though rare, selected cases of both posterior fossa dermoid tumors and teratomas have been reported in patients with KFS, with the majority of such masses being histologically benign [2].

Teratomas are germ cell neoplasms composed of tissues derived from all three embryological germ layers. Teratomas can be classified as either mature or immature, with mature teratomas considered benign tumors given low to absent mitotic activity, and characterized histologically by fully differentiated endoderm, mesoderm, and ectoderm. Immature teratomas, by contrast, constitute 10–50% of all teratomas and are commonly malignant [3, 4].

Teratomas of the head and neck are quite rare and generally present during the neonatal period, and while pediatric teratomas tend to be benign, in the adult these tumors are typically malignant [5]. Intracranial teratomas account for approximately 0.3–0.9% of all brain tumors and mimic other intracranial germ cell tumors in their tendency to present in midline sites, like the pineal gland and suprasellar regions [4, 6]. Benign teratomas arising from the midline nasal septum have been well described and resected endoscopically [4, 7]; however, a skull base teratoma on a neurovascular stalk arising from the craniopharyngeal duct, superimposed on a KFS background, presents a singular challenge. To resect a pedunculated mass with these features, combined ultrasonic and bipolar diathermy was used to cross-clamp and ligate the pedicle without concern for bleeding from, or retraction of, an uncontrolled skull base pedicle.

## 2. Case Report

**Figure 1:**
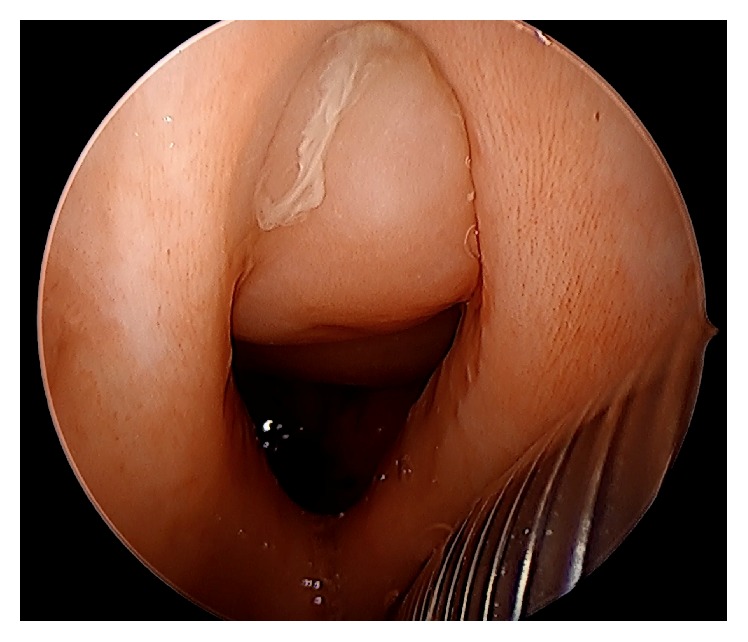
Endoscopic view of teratoma extending into the oropharynx. Transoral endoscopic view of a large mucosalized mass extending from the nasopharynx into the upper aspect of oral cavity and pharynx and occluding the cleft palate defect. The metal rings represent the reinforced endotracheal tube placed transorally following induction of anesthesia.

A 38-year-old female presented with a large posterior cavity nasal mass that was being expectantly observed by outside physicians for years. However, on presentation, she endorsed worsening headaches, troublesome nasal obstruction affecting sleep, and intermittent nausea and vomiting without associated photophobia. Physical examination and neck radiograph imaging suggested a diagnosis of KFS with the patient exhibiting webbing of the neck, cleft lip and palate, and midline cranial and cervical anomalies including C2/C3 cervical fusion. Office endoscopy revealed a complex, mobile, midline mucosalized mass filling the entire posterior nasal airway with extension into the superior oropharynx through a cleft in the soft palate (Figure 1).

**Figure 2:**
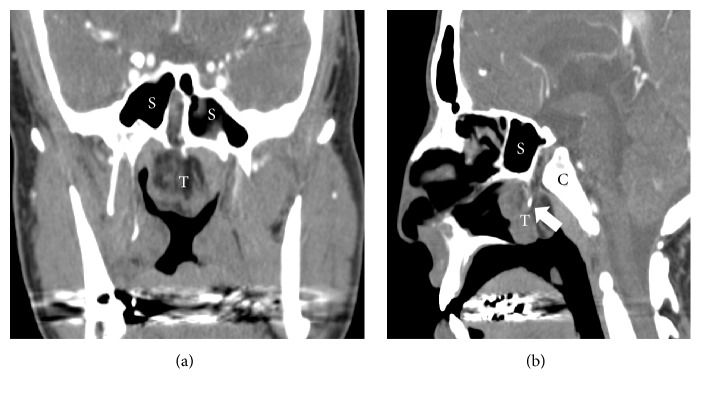
CT angiogram (CTA) imaging of pedunculated skull base lesion. (a) Coronal CT slice at level of the bilateral sphenoid sinuses (S), with tumor pedicle arising between sinus cavities, at typical site of intersinus septum. The 4 × 4 cm tumor (T) is composed of heterogeneously dense material, with suspected low-density (dark) fat seen. (b) CTA in sagittal view, showing relationship of tumor pedicle extending between anterior sphenoid sinus (S) and posterior clivus (C). Arrow shows the diminutive artery coursing through the pedicle, without direct communication with the ICA and vertebrobasilar system (not shown).

**Figure 3:**
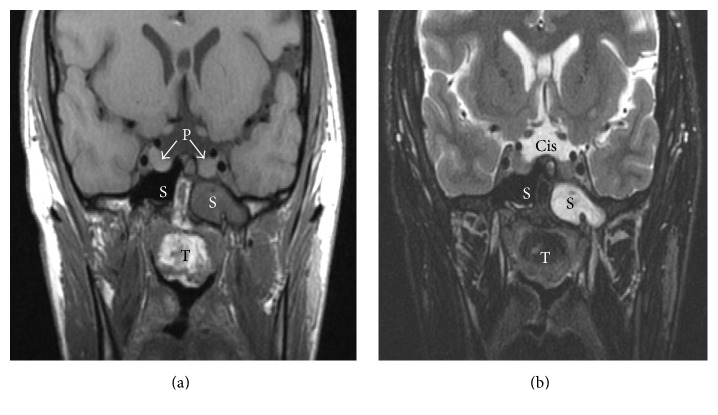
Magnetic resonance imaging (MRI) of pedunculated skull base lesion. (a) Coronal T1 MRI clearly demonstrates the tumor extending from intracranial midline defect into the nasopharynx. The bright intensity signal within the tumor (T) and stalk between the sphenoid sinuses (S) on T1 sequence represents fat, nearly pathognomonic of teratoma. Pituitary duplication (P with arrows) is best appreciated on T1 imaging as well. Of note, the left sphenoid sinus was noted to have mucosal thickening at the time of MRI compared to CT imaging (Figure 2). (b) Coronal T2 sequence at same slice location highlights the enlarged midline basilar cistern (Cis) with hyperintense signal representing cerebrospinal fluid.

Computed tomography angiography (CTA) revealed a 4 × 4 cm multiloculated, nasal mass of the sphenoid skull base protruding through a “central corridor” in the sphenoid intersinus septum of the basisphenoid and anterosuperior to the clivus bone (Figures 2(a) and 2(b)). Magnetic resonance imaging (MRI) demonstrated a pedunculated, extradural heterogeneous mass extending into the nasopharynx, with high intensity fat signal seen within the tumor and its sizeable stalk on T1 sequence (Figure 3(a)). Rare pituitary duplication (Figure 3(a)) and the enlarged intracranial basilar cistern with bright CSF fluid signal are readily noted on T2 sequence (Figure 3(b)). The tumor stalk was felt to represent a patent/persistent craniopharyngeal duct, and multiple other midline intracranial abnormalities including corpus callosum dysgenesis, midline lipoma, and dysmorphic hypothalamic and brainstem changes were also noted (not shown). CTA was also performed out of concern for large caliber vascular pedicle to the tumor, with only limited axial blood supply noted (Figure 2(b)). Given the patients' crescendo in symptoms and the collective imaging findings, an extended endonasal resection of this skull base teratoma was planned between otolaryngology and neurosurgery.

**Figure 4:**
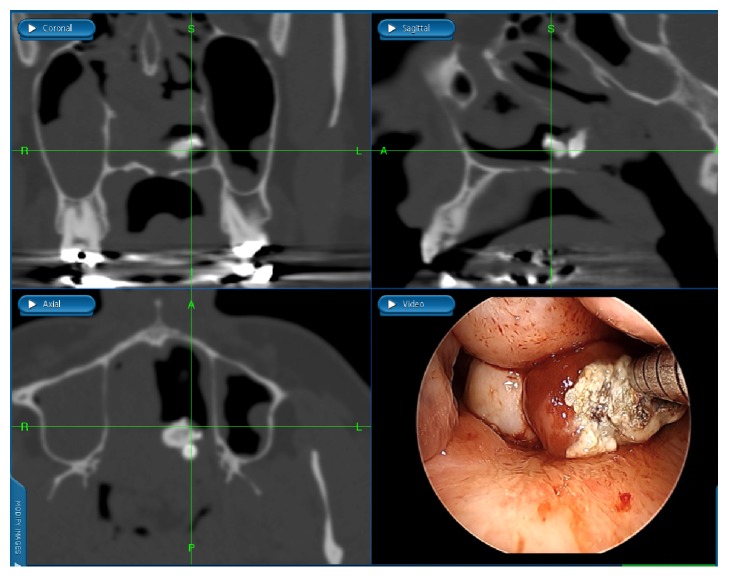
Use of intraoperative CT image guidance assistance during skull base approach. Computer-assisted navigation system (image guidance) highlighting intraoperative CT scan imaging in coronal, sagittal, and axial views. With the registered instrument tip contacting the anterior face of the lesion (endoscopic image at bottom right), the fine cut maxillofacial CT scan shows calcified material present within the skull base teratoma at this site of tumor contact (intersection point of green lines), representing intratumoral dentition.

**Figure 5:**
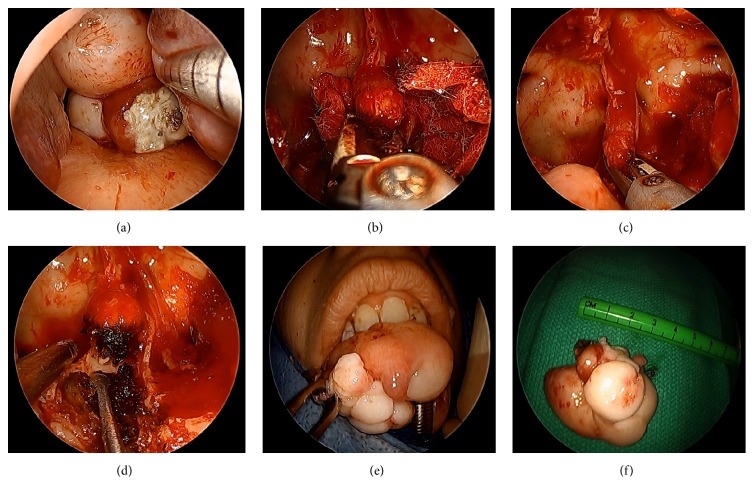
Surgical resection of skull base teratoma with ultrasonic bipolar diathermy. Endonasal view of the pedunculated skull base teratoma obstructing the posterior nasopharynx, with metal suction tip retracting the left inferior turbinate (a). Following sphenoidotomies, the tumor pedicle is approached (b), clamped in a full-thickness manner (c), and completely transected (d) using the vice-clamp tip and combined ultrasonic/bipolar platform energies. Teratoma passed into the oral cavity for transoral en bloc tumor removal (e), with lesion measuring approximately 4.0 cm in diameter (f).

**Figure 6:**
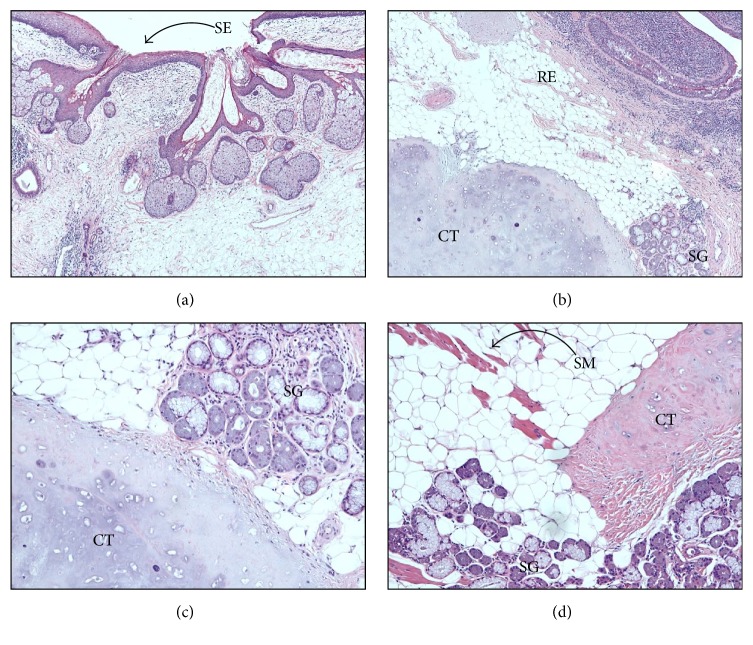
Representative histopathology photomicrographs of the skull base teratoma. (a) Stratified squamous epithelium (SE) with associated adnexal structures (4x magnification). (b) Ciliated respiratory epithelium- (RE-) lined cystic structure, salivary gland tissue (SG), and cartilage (CT) (4x magnification). (c) Scattered foci of SG adjacent to CT tissues (10x magnification). (d) Haphazard arrangements of CT, SG, and striated muscle (SM) (10x magnification). Mature adipose tissue is also present in the background (unlabeled clear cell bodies).

Access was obtained via inferior turbinate outfracture and limited posterior septectomy to permit binarial access. A two-surgeon, four-handed surgical approach, and intraoperative, computer-assisted image guidance confirmed unfettered access to the teratoma, with classic dentition seen in triplanar view (Figure 4). The main consideration was control of the midline stalk at the skull base without encountering a cerebrospinal fluid (CSF) leak from the basilar cistern or intracranial retraction of vascular feeders from the pedicle. The stalk, measuring approximately 1 cm laterally and 1.5 anteroposteriorly, was encased in a bony shell that extended through the midline floor of the sphenoid sinuses into the upper clivus. Using hand instruments and fine diamond drill bits, the ensconced stalk was liberated from the surrounding bone. This revealed a fibrovascular pedicle that was then cleanly truncated using the Thunderbeat™ device, for simultaneous ultrasonic wave (cutting) and bipolar diathermy (coagulation) action. The technique permitted transmural pedicle resection, precluding the need for awkward suture ligation of a thick pedicle adjacent to the skull base, while allowing for the tumor to be delivered en bloc transorally (Figure 5). Microscopic analysis of the resected tumor using standard histology revealed a disordered arrangements of mature epithelial and mesenchymal tissues, including cartilage, adipose tissue, stratified squamous epithelium with cutaneous-type adnexal structures, ciliated respiratory-type epithelium, and striated muscle (Figure 6). No immature neural element was identified, consistent with a mature teratoma. No perioperative complications were noted, with resolution of all preoperative symptoms within 1 month. Postoperatively, in the absence of the obstructive mass, a palatal obturator was required to limit nasal regurgitation and hypernasal speech through coverage of the cleft palate defect. She continues to do well >3 years since surgery, with no untoward sequelae from the procedure.

## 3. Discussion

Teratomas are germ cell tumors that classically recapitulate all embryonic cell lines: endoderm, mesoderm, and ectoderm. They can be further classified as mature, immature, or malignant [8]. Mature teratomas are well-differentiated, generally benign masses possessing locally aggressive behavior (adhesion to/displacement of adjacent structures) [9]. Head and neck sites are rare and represent only 2% of all teratomas [5]. CT and MRI imaging are essential to determine lesion characteristics, such as the presence of mixed density tissues such as fat, muscle, bone, soft tissue, and cartilage. Suprasellar teratomas can be particularly challenging as the pituitary infundibulum can appear convoluted or elongated from local mass effect [9]. Such lesions that involve the skull base have been previously removed using an endoscopic endonasal approach [10]. Here, we report a striking case of a female patient with KFS with a longstanding, pendant skull base teratoma extending through a patent craniopharyngeal duct leading to obstruction of the nasopharynx and worsening headaches. While CT imaging showed a posterior nasal mass protruding through a defect in the clivus, MRI revealed an extradural mass containing admixed dentigerous and adipose pockets extending from the middle cranial fossa through the basisphenoid with associated dramatic duplication of the pituitary gland and vertebrobasilar feeders. This case highlights our experience with the workup and treatment of challenging skull base pathology and also the innovative intranasal utilization of the Thunderbeat device for management of the extradural stalk through an endoscopic approach. Postoperative examination of the mass revealed a benign, mature teratoma.

Patients with KFS can have anatomic abnormalities such as vertebral fusion, cleft palate, aortic arch anomalies, renal agenesis, spina bifida, and cerebral structural abnormalities [2]. This patient presented with cervical vertebral fusion, cleft lip and palate, webbed neck, anatomic duplication of the basilar artery and pituitary gland, midline developmental intracerebral dysmorphia, and patent craniopharyngeal duct. The association between posterior fossa dermoid tumors and KFS is well established, with approximately 24 reported cases in the literature [2, 3]. A teratoma arising from the middle cranial fossa at the skull base, however, has not been reported. A patient with both KFS and a middle cranial fossa skull base teratoma presents a unique challenge in terms of both nonsurgical and surgical management due to anatomical variation and possible endocrine abnormalities. To our knowledge, this is the first case in the literature that highlights the management of both of these two rare disease processes.

The preoperative assessment of patients with either sellar or suprasellar teratomas includes otolaryngologic, endocrine, ophthalmologic, and neurological evaluations. Appropriate endocrine studies may suggest signs and symptoms of diabetes insipidus and anterior hypopituitarism [11], but lab studies of serum pituitary hormone levels and related functional studies were unremarkable in this patient, as were formal visual field and acuity testing. Although contrasted MRI assists in the evaluation of intracranial masses, this imaging confirmed intranasal extension through the skull base and strongly suggested the final diagnosis of teratoma. As in this case, treatment of mature teratomas is primarily surgical and affords a low recurrence rate following complete extirpation [11]. Endoscopic endonasal resection of skull base lesions is increasingly common, but to our knowledge, this is the first report of endoscopic resection of a skull base teratoma in the setting of pituitary duplication through use of combined ultrasonic bipolar diathermy.

The endonasal approach undertaken with neurosurgery allowed for complete, single stage tumor resection without CSF leak or pedicle hemorrhage or retraction. Given the unusual pendant teratoma on a dense fibrofatty stalk, complete pedicle control and transmural transection at this deep skull base site was mandatory, while avoiding suture ligation. Using the clamp on the Thunderbeat device to grasp the pedicle transnasally, the tumor stalk was able to be cleanly transected using the combined ultrasonic and bipolar energy sources transmitted through the “teeth” of the clamp, with minimal eschar and heat transmission [12, 13]. The dual modality ultrasonic bipolar instrument described allowed us to deftly manage the wide, thick, and potentially hemorrhagic tumor pedicle to this unusual skull base mass. In this case, single instrument ligation and hemostasis allowed for endoscopic resection with minimal blood loss and minimal risk of complications.

Skull base teratomas have been well associated with anatomic variants such as pituitary gland duplication, which typically warrants additional neuroendocrine workup. We identified approximately 41 documented cases of pituitary duplication in our literature analysis. Of these reports, less than half of cases are associated with the presence of a skull base teratoma [14–16]. Common anomalies associated with pituitary gland duplication include vertebral anomalies and cleft palate [16], both of which are present in this patient. Pituitary gland duplication is an exceedingly rare malformation that aligns with other midline craniofacial anomalies involving the skull base, midline developing notochord, and pharynx [15, 17], although the basis for this is not well understood. The cleft created by ultimate separation of the prechordal plate and notochordal process is theorized to lead to a potentially sizeable skull base defect as noted in this patient [15].

Surgical management of sinonasal teratomas is primary treatment, and naturally given the complex patient history and potential for comorbidities, a multidisciplinary team between otolaryngology, neurosurgery, and endocrinology was involved in the surgical and postoperative management of this patient. Benign, mature teratomas have been reported to have survival rates over 90% after 10 years [9]. However, tumor recurrence has been reported through a phenomenon known as “growing teratoma syndrome,” which is extremely rare and refers to a relapse of malignancy due to partial response to surgical resection or chemotherapy [18]. In this syndrome, the recurrent tumor is often resistant to chemotherapy and radiation but generally only occurs in those with primary malignant tumors [18]. In this case, the patient had a benign, mature teratoma with no evidence of recurrence 3 years following removal. Contributing to the lack of tumor regrowth is likely near-total truncation of the skull base pedicle, which allowed for complete surgical resection of the teratoma mass. This could be achieved in a reassuringly atraumatic and hemostatic manner using the described combined cutting/coagulating technology in a novel application.

## 4. Conclusion

The typical treatment for benign, mature teratomas causing symptoms is surgical resection, which was successfully performed in this case involving a skull base teratoma extending from the skull base and middle cranial fossa. A multidisciplinary team approach is recommended in these cases due to the complexities of the disease process, aberrant anatomy, and potential for complications. In a patient with KFS and the presence of pituitary duplication and cleft palate, the use of ultrasonic bipolar diathermy allowed for complete and bloodless control and transection of the dense skull base pedicle and gratifying en bloc resection of the mass through the oral cavity. Future reports on the use of this dual modality technology in endonasal procedures and skull base surgery will help ascertain its broader utility and impact on outcomes.
